# Rethinking MRI Protocols for Pituitary Microadenomas: Prioritizing Non-Contrast Imaging for Safe Follow-Up

**DOI:** 10.3390/tomography11090105

**Published:** 2025-09-12

**Authors:** Fariba Zarei, Farideh Nematollahi, Asadolah Jalil, Banafsheh Zeinali-Rafsanjani, Mahdi Saeedi-Moghadam

**Affiliations:** 1 Medical Imaging Research Center, Shiraz University of Medical Sciences, Shiraz 71936-35899, Iran; zareifari@gmail.com; 2 Department of Radiology, Shiraz University of Medical Sciences, Shiraz 71936-35899, Iran; f_nematollahi@yahoo.com (F.N.); asadjalil1360@gmail.com (A.J.)

**Keywords:** pituitary adenoma, dynamic MRI, non-contrast MRI, gadolinium, microadenoma

## Abstract

Introduction and Objectives: Dynamic contrast-enhanced magnetic resonance imaging (DCE-MRI) has been used as a gold standard in diagnosing and following pituitary microadenomas. However, the use of gadolinium-based contrast agents (GBCAs) involves a potential risk of long-term retention in tissues and adverse reactions. This study aimed to evaluate the sensitivity of non-contrast MRI (T1W and T2W sequences) in follow-up imaging of pituitary microadenomas, attempting a comparison with DCE-MRI, assessing tumor stability over time. Materials and methods: We retrospectively reviewed 300 pituitary MRI scans between 2020 and 2024. Included were patients with confirmed microadenomas (≤10 mm). Non-contrast (T1W/T2W) and DCE-MRI sequences were analyzed by an experienced radiologist blinded to any clinical information. Detection rates and changes in tumor size were evaluated. Results: Detection rates for 79 microadenomas were 55.7% for T1W, 70.9% for T2W, and 88.6% for DCE-MRI. There was no significant tumor growth during the follow-up (mean size 4.80 ± 2.3 mm vs. 4.81 ± 2.4 mm, *p* > 0.5). Conclusions: While still more sensitive for the primary diagnosis, the non-contrast MRI was able to visualize the majority of detected microadenomas, and significant growth was ruled out, thus supporting the case to omit gadolinium from follow-up imaging in stable cases. This may translate to lower costs and decreased patient risk from contrast-related hazards.

## 1. Introduction

A pituitary adenoma is the most common tumor within the sellar and parasellar regions [1,2]. These benign tumors have important effects on hormonal balance and neurological functions, with a wide range of clinical presentations such as headaches, visual disturbances, and endocrine disorders, including hyperprolactinemia and Cushing's disease [3,4,5]. Accurate diagnosis and follow-up of the case of pituitary adenoma are very important, considering that appropriate and timely intervention may prevent complications arising out of tumor growth and hormonal disturbance [6].

Different modalities can be used to detect and follow the pituitary lesions such as computed tomography (CT), positron emission tomography (PET), and magnetic resonance imaging (MRI). CT gives the best bony detail of the sella turcica and is inferior to MRI in looking at the soft tissues. The PET scan, especially using gallium-68 DOTATATE, has shown promise in the detection of hormonally active adenomas but is limited in use due to its expense and limited availability. Hence, MRI, with its superior soft tissue contrast and free of radiations, carries paramount importance in pituitary imaging [7,8].

MRI has emerged as the gold standard for the diagnosis of pituitary adenomas, given its unique soft tissue contrast and detailed delineation of small structures [9,10]. Dynamic contrast-enhanced MRI, in particular, has gained wide acceptance for initial diagnosis and follow-up of these tumors. Recent smaller studies have questioned the usefulness of contrast for follow-up imaging. Hordejuk et al. (2023) described stability in microadenomas with infrequent imaging, while Constantinescu et al. (2024) have promoted less stringent follow-up protocols for non-functioning microadenomas [11,12]. This imaging technique is very sensitive and specific, thus enabling clinicians to outline the adenomas as focal areas with reduced enhancement compared to the surrounding pituitary parenchyma. Dynamic MRI tends to increase the detection rates for microadenomas that are generally not visible on non-contrast images. Conventional non-contrast MRI sequences, such as T1W and T2W scans, may also reveal some adenomas but are perhaps less sensitive in the detection of small lesions [13,14,15].

However, despite the diagnostic superiority of contrast-enhanced MRI, safety concerns regarding gadolinium retention have emerged concerning the use of gadolinium as a contrast agent. Life-threatening complications include nephrogenic systemic fibrosis and accumulation in the brain after repeated administration; thus, it raises the concern of the need for reevaluation of the indication of gadolinium, especially for follow-up examination in patients with microadenomas [16,17,18]. Awareness of gadolinium retention has increased, and because to this, recommendations on the judicious use of contrast agents have been made, particularly for patients with renal impairment or those requiring multiple follow-up scans [19,20,21].

While dynamic contrast-enhanced MRI constitutes the current gold standard in the diagnosis of pituitary microadenoma, the known risk of gadolinium retention necessitates reassessment of its usage in follow-up. The literature has failed to come to a consensus on whether non-contrast MRI is appropriately able to monitor microadenomas without compromising their diagnostic accuracy. While current guidelines (American college of radiology (ACR), 2021; European academy of neurology (EAN), 2023) consider DCE-MRI for initial diagnosis, no particular follow-up protocol is recommended for stable microadenomas. We formulate the hypothesis that non-contrast MRI (T1W/T2W sequences) provides sufficient sensitivity to follow-up stable pituitary microadenomas that the administration of gadolinium-based contrast might well be omitted in these cases without departing from the high standards of diagnostic accuracy. This study, therefore, sets out to compare non-contrast and dynamic MRI in the sensibility of microadenoma detection and to evaluate changes in size over time for safe omission of gadolinium in follow-up protocols. Our hypothesis determines that non-contrast MRI alone may suffice for monitoring stable microadenomas while exposing such patients to fewer risks involving contrast administration.

## 2. Materials and Methods

### 2.1. Study Design

In this study, conventional non-contrast MRI was compared with dynamic contrast-enhanced MRI for diagnosing pituitary microadenomas using a retrospective cross-sectional design. This study was performed in compliance with relevant ethical considerations and gained the needed approval from the Faculty Research Ethics Committee of Shiraz University of Medical Sciences under the Ethical Approval Code: IR.SUMS.MED.REC.1397.466; date: 21 January 2019.

**Figure 1:**
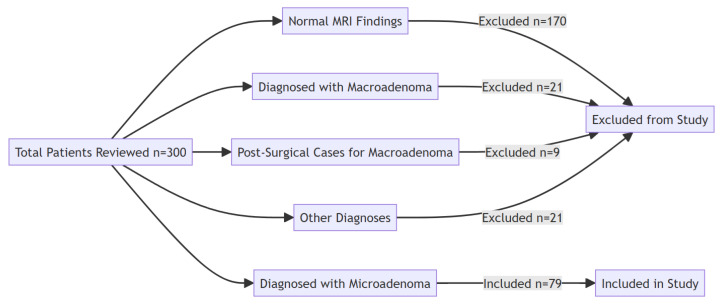
Flowchart illustrating patient selection for this study.

The selection of patients was carried out by reviewing MRI studies of patients who underwent imaging of the pituitary region from January 2020 to December 2024. Inclusion criteria included patients diagnosed with pituitary microadenoma, defined as a focal area measuring ≤ 10 mm on MRI. For this study, only such patients whose MRI studies—both non-contrast and dynamic contrast-enhanced MRI—were available for scrutiny were considered, regardless of whether microadenomas were functional or non-functional. Conversely, all patients with macroadenomas, other forms of pituitary lesions, or cysts were excluded, as were patients whose MRI scans showed poor quality or significant artifacts. In addition, patients whose medical records were incomplete and did not have follow-up MRI data were excluded from consideration (Figure 1).

### 2.2. MRI Protocol

MRI scans were performed on a 1.5 Tesla (T) Philips scanner (Philips Medical Systems, Best, The Netherlands). A 1.5T scanner was used since it represents >75% of global pituitary MRI capacity (WHO 2023), to avoid 3T-specific susceptibility artifacts in the sella, and because it matches the field strength used in most prior pituitary microadenoma studies [22,23]. The non-contrast MRI protocol included T1-weighted (T1W) and T2-weighted (T2W) images in both sagittal and coronal planes. The pulse sequence parameters of T1W, T2W, and DCE-MRI are provided in Table 1. Fat suppression was not applied. The non-contrast scan takes about 15 min.

In the case of contrast-enhanced imaging, gadobutrol (Gadovist®, 1.0 mmol/mL; Bayer AG, Leverkusen, Germany) was used at a dose of 0.1 mmol/kg body weight, which, for a 70 kg patient, was 7 mL, administered as a bolus intravenous injection, followed by a 20 mL saline flush. Gadobutrol is a macrocyclic GBCA and was chosen based on the ACR/National Kidney Foundation (NKF) international safety guidelines due to its high thermodynamic stability and very low risk of nephrogenic systemic fibrosis (NSF). Dynamic imaging started immediately after the injection of contrast, acquiring six series of coronal images at different time points (0, 30, 60, 90, 120, and 180 s) to evaluate the enhancement pattern. Post-contrast images were also acquired in both coronal and sagittal planes using the same parameters as the non-contrast sequences and the scan duration was almost 10 min.

Subsequent MRI follow-ups were carried out over 6–12 months’ intervals. For each patient, between 1 and 6 scans were evaluated with a mean of 2.2 ± 1.1. The radiologist remained blind to prior diagnoses during the follow-up size assessment.

### 2.3. Data Collection

Scans were reviewed by a neuroradiologist with 12 years of pituitary MRI experience. The radiologist was blinded to the clinical details and previous imaging results to avoid bias. The reviewer had to assess the presence and size of microadenomas on both non-contrast and dynamic MRI and compare the sensitivity of both imaging modalities. For this, sensitivity was considered as the ratio of true positives regarding microadenomas found with each methodology. Moreover, the change in size in subsequent MRI is observed; for that purpose, the size is measured on its largest diameter using suitable tools in the software.

All scans were first clinically assessed by the onsite radiologists. For the purposes of this study, a second radiologist with 12 years’ of experience in pituitary MRI, retrospectively and independently read all cases while being blinded to original clinical reports, patient identifiers, and chronological order of follow-up scans. Intra-rater reliability was also assessed by re-reading a randomly selected 20% subset (*n* = 16) after a 4-week interval, and the agreement was excellent (κ = 0.86 for lesions detected, ICC = 0.92 for size). Inter-reader consistency was evaluated by comparing the interpretations of the research radiologist, who was blinded to all clinical data, with original clinical reports from onsite radiologists. There was an 87% concordance (κ = 0.78) in lesion detection, a mean difference of 0.3 ± 0.4 mm (ICC = 0.89) in size measurement, and a 92% agreement for definitive calls (Likert ≥4). There were 9 discrepancies, which were resolved through consensus review with a third senior neuroradiologist.

### 2.4. Statistical Analysis

The analysis was conducted using SPSS version 21 (IBM Corp., Armonk, NY, USA). Results are presented as means ± SD for continuous variables, whereas frequencies and percentages were used for categorical variables. The chi-square test was used for comparison of the sensitivity of non-contrast MRI and dynamic MRI, and *p* < 0.05 was considered significant. Power analysis, subsequent to data collection, and conducted using the chi-square test in GPower 3.1 software, indicated a power of 92% to detect a medium effect size (Cohen’s w = 0.35) at α = 0.05, thus confirming the sufficiency of the sample size (*n* = 79) for comparative sensitivity. W = 0.35 was chosen because it closely matches our calculated effect (w = 0.33), it exceeds literature values (Indrajit 2001 [23]: w = 0.30), and it aligns with the >15% sensitivity difference that changes clinical practice [24].

Mean sizes of microadenomas at the time of first and follow-up MRI examination were compared by the *t*-test to identify if any change in the size occurred in a significant manner over the period of time. Normality expectations were validated by Shapiro–Wilk tests (all *p* > 0.15), Q-Q plots, and skewness (−0.3 to −0.4). While it is common to recommend nonparametric methods when the sample size (*n*) is small, our sample size of *n* = 79 and the normal distribution of differences allow us to utilize *t*-tests confidently.

## 3. Results

Of the 300 MRIs studied, 50 (16.7%) were males and 250 (83.3%) were females. The average age of the patients was 32 ± 11.5 years for both sexes, with an age range of 8 to 77 years old.

Among the 300 patients, normal MRI findings were reported in 170 (56.7%) patients, 79 (26.3%) patients were diagnosed with microadenoma, 21 (7%) patients had macroadenoma, and 9 (3%) patients were under follow-up post-surgery for macroadenoma. The rest, 21 (7%) patients, had other diagnoses like cysts, brain tumors, etc. (Figure 1). Therefore, 79 patients with microadenoma were included, and the others were excluded from this study. Our analysis revealed that there were not clinically meaningful differences between the included (*n* = 79) and excluded subjects (*n* = 221) in terms of age (33.2 ± 12.1 vs 34.5 ± 11.7 years (*p* = 0.38)) and sex (89.9% vs 85.5% female (*p* = 0.32)).

Of 79 microadenomas, 71 (89.9%) occurred in females and 8 (10.1%) in males, with a mean age of 33 ± 12 years. Of the 79 patients with microadenoma, as identified in dynamic images, 44 patients (55.7%) were visible in T1W sequences, 56 patients (70.9%) in T2W sequences, and 70 patients (88.6%) in postcontrast (post-gad) sequences. The chi-square test proved that the sensitivity of post-contrast sequences was significantly higher (88.6%) than that for T1W (55.7%, *p* < 0.001) and T2W (70.9%, *p* = 0.005). These findings are summarized in Table 2, which outlines the detection rates across different MRI sequences.

The mean largest size of the microadenomas at the first and last MRI was 4.80 ± 2.3 mm and 4.81 ± 2.4 mm, respectively, which was not significantly different (*p* > 0.5) by T-test. Bland–Altman analysis of tumor size measurements (baseline and last follow-up) showed a mean difference of 0.01 mm (95% CI: −0.12 to +0.14 mm), and limits of agreement of −0.38 mm to +0.40 mm.

Of the 79 patients, 34 (43%) had one MRI, and 45 (57%) had two or more MRIs in the given timeframe. This includes 31 patients (39.2%) who had two MRIs, 8 with three MRIs (10.1%), 4 with four MRIs (5.1%), and 1 with five and six MRIs (1.3%). Of the 170 patients whose MRIs were reported as normal, 21 patients (12.4%) had two MRIs, and 3 patients (1.8%) had three MRIs within the four years despite their normal MRI results.

Among 10 such cases of microadenoma, onsite radiologists who had reported the follow-ups prior to this study identified some changes in tumor size, but the expert radiologist in this study who reviewed all MRIs reported no such changes, and size discrepancies were related to measurement techniques and/or the lack of attention to coronal images. It is worth noting that of those 10 cases with prior discrepant size measurements, mean absolute difference was 1.2 ± 0.4 mm (range: 0.5–2.1 mm); in 70% of cases, onsite radiologists reported larger sizes (mean +1.3 mm), and in 30%, they reported smaller sizes (mean −0.9 mm).

Although, the main cohort contained patients referred between 2020 and 2024, Figure 2, Figure 3 and Figure 4 shows a representative case with historical scans (2015–2020), retrieved from PACS, to demonstrate temporal stability. No evidence of recurrence of tumors in any of the nine who underwent resection of macroadenomas during this period was seen.

Stratification showed a pronounced peak of microadenoma prevalence among reproductive-age women; 68.4% of cases (54/79) were seen in the 25–45 years age range. The distribution was quite significantly different between men and women; 87.3% congregated in the 25–45 range, with a peak incidence between 30 and 39 years (40.8%). Prolactinomas were two pediatric cases (2.8%, ages 8 and 15). In contrast, microadenomas in men (*n* = 8) were almost evenly distributed, ranging from 20 to 60 years (median 42, IQR 28–53), with no cases below the age of 20. This distribution pattern was significantly different per sex (*χ*^2^ = 14.2, *p* < 0.001), thereby reinforcing a hormonal influence in adenomagenesis [25,26].

## 4. Discussion

The pituitary gland plays a major role in the regulation of various metabolic processes and the growth of the body. Pituitary adenomas are considered to be the most common tumors in the sellar and parasellar regions, arising from the cells of the anterior lobe of the pituitary gland. Previous studies have indicated that the prevalence of these tumors puts into significance the need for appropriate diagnostic imaging techniques [22,23]. Currently, MRI with contrast injection is the modality of choice for the imaging of the pituitary gland, the detection of masses, and follow-up in patients with adenomas. There is recognition of a dynamic imaging sequence as the best MRI strategy to evaluate pituitary adenoma since it shows greater sensitivity to small alterations in tumor size and morphology [10,22,24].

Our study results emphasize the crucial role of dynamic contrast-enhanced MRI in the identification and treatment of pituitary microadenomas. As our results show 88.6% detection of microadenoma in postcontrast sequences (Table 2), there is a requirement for state-of-the-art MR techniques to improve the accuracy of the diagnosis.

A gender disparity was evidenced from our analyses of 71 females and 8 males diagnosed with microadenomas. They correlate with previous findings of a higher female prevalence of pituitary adenomas [27,28]. The increased gender difference in incidence rates raises a question about the biological and hormonal mechanisms leading to such a difference.

There is evidence that hormonal mechanisms are likely to play a very important role, and among them, the role of estrogen is paramount in the progression and development of pituitary adenomas. Estradiol seems to exert an effect on the proliferation of pituitary cells and thus may increase the risk of tumor formation in females. Some studies have indicated that estrogen promotes the growth of some kinds of pituitary particularly prolactin-secreting tumors, which just happen to be more common in women. This same hormonal influence is supported once more by our findings since, as mentioned above, the majority of our patients were females, suggesting an association of estrogen in microadenoma development [25,26].

Our observed age distribution, with 68.4% of the microadenomas appearing in women aged 25–45, and a peak incidence rate between 30 and 39 years, highlights the pivotal role of hormonal signaling in pituitary tumorigenesis. These trends correspond to estrogen-dependent pathways of proliferation, particularly for prolactinomas constituting most of the microadenomas in reproductive-age women [25,26]. On the contrary, the absence of clustering in any specific age group among male patients (clinically symmetrically distributed over 20–60 years) provides evidence for sex-differentiated etiologies. These data emphasize the need for sex-related surveillance systems, with particular emphasis on premenopausal women harboring incidental microadenomas. Hormonal activity could indeed modulate the progression of these tumors.

Understanding this gender difference is crucial in developing specific management approaches. For instance, understanding that females are more likely to be affected by pituitary adenomas can enlighten clinicians as to how to use screening and diagnostic methods effectively to ensure that women receive appropriate assessment, especially during their reproductive years [3,7]. In addition, the impact of these tumors on reproductive health should not be overlooked. Healthcare practitioners should be aware of the potential fertility issues along with hormonal disturbances that could arise due to microadenomas. In particular, prolactinomas are associated with elevated levels of the hormone prolactin, which might lead to menstrual dysfunction and infertility issues in women [29,30,31].

Considering these aspects, there is a need for a multidisciplinary approach. There might be a need for consultations with endocrinologists or reproductive specialists regarding the management of hormonal imbalances associated with pituitary adenomas. These specialists can thus offer targeted interventions, such as pharmacological interventions, to balance the hormonal state or methods that can help preserve fertility in those affected [32,33]. It is also important to understand the psychosocial implications of such diseases on female fertility, as the diagnosis of a pituitary adenoma could lead to increased anxiety and concerns about fertility and hormonal health [34].

The increased use of gadolinium-based contrast media has raised significant concern regarding their safety profile in clinical practice. While these agents are important in enhancing the detection of pituitary adenomas during MRI, one must be aware of the risks associated with their use. Adverse hypersensitivity reactions may occur, ranging from mild allergic reactions to severe life-threatening anaphylaxis, hence being an immediate danger to the health of a patient. Nephrogenic systemic fibrosis is also another important medical condition related to gadolinium exposure, which is observed in patients who have weak renal function. The onset of nephrogenic systemic fibrosis (NSF) often leads to severe symptoms, such as thickened and hardened skin, and affects several systems of the body, pointing out a dire need to be more cautious while using the element on vulnerable groups [35,36,37].

**Figure 2:**
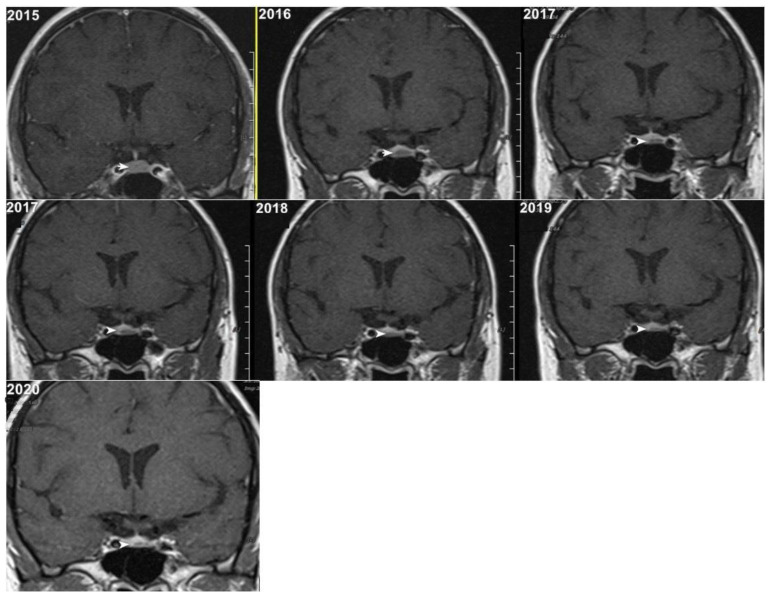
Representative coronal images (2015–2020) of a stable microadenoma (arrows) of a 33 Y/O woman. The left upper image from 2015 is a delayed image, and the other six images are dynamic MRI follow-ups for this patient. In 2017, the patient had two follow-ups in the first and second half of the year. In the 5 years of follow-up, no size change can be seen.

**Figure 3:**
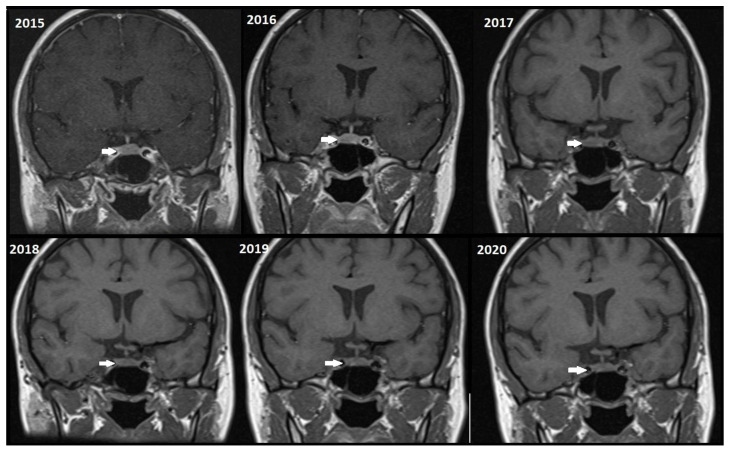
Representative T1W coronal images (2015–2020) of a stable microadenoma (arrows) of a 33 Y/O woman. In the 5 years of follow-up, no size change can be seen.

**Figure 4:**
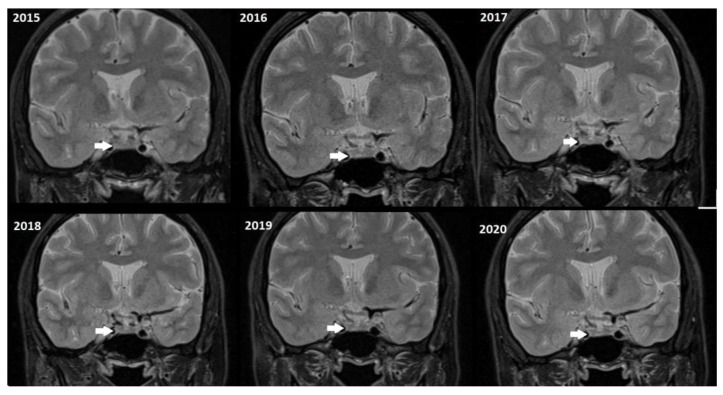
Representative T2W coronal images (2015–2020) of a stable microadenoma (arrows) of a 33 Y/O woman. In the 5 years of follow-up, no size change can be seen.

Moreover, repeated exposure to gadolinium is associated with the deposition in basal ganglia and the dentate nuclei of the cerebellum. This deposition results in an increased signal intensity seen in T1-weighted imaging that has raised concerns concerning potential long-term neurological effects. These safety concerns are thus especially important in the case of patients with microadenomas who may require repeated MRI surveillance [38,39]. Given the fact that patients with microadenoma may be exposed more frequently to MRI with contrast, our study investigated conventional MRI and dynamic imaging for diagnosis and follow-up in patients with pituitary microadenomas undergoing MRI at our institution. We also evaluated the change in size of microadenomas in follow-up MRI studies and the necessity of injecting contrast media in monitoring this set of patients (Figure 2, Figure 3 and Figure 4).

The vast difference in sensitivity between these imaging techniques-55.7% for T1-weighted images and 70.9% for T2-weighted images confirms the superiority of contrast-enhanced MRI with 88.6% sensitivity (Figure 1). This was in line with previous studies [22,23,40]. The study of Bashari et al., reported dynamic contrast-enhanced MRI is more sensitive to detect microadenomas, while T2W images can help visualize microadenoma, which may appear hyperintense on T2-weighted images, potentially reducing the need for gadolinium enhancement. The authors declared that T2W can also provide valuable supplementary information in specific clinical settings, thus furthering diagnosis and aiding in treatment planning through enhanced accuracy [24]. Jipa et al., report that dynamic MRI performs better in assessing enhancement patterns of lesions, thus assisting in the diagnosis of microadenomas. They added that T1-weighted post-contrast sequences are useful in differentiating pituitary adenomas from surrounding tissue. They also noted the inadequacies of non-contrast T2-weighted sequences for microadenoma detection in comparison to dynamic or post-contrast images [41].

Our T1-weighted detection rate (55.7%) parallels the literature on non-contrast sequences limitations. Bashari et al. [24] report that the normal anterior pituitary is isointense with gray matter on T1W/T2W imaging which contributes to the difficulty in detection of microadenoma in the absence of contrast. Farabola et al. [22] reported that T1W+T2W spin-echo combined sequences were only able to identify 64% of microadenomas and reported sensitivity of 80% with dynamic MRI—a similar pattern to our data (DCE-MRI: 88.6%). Notably, 16% of Farabola’s microadenomas were detected only by dynamic imaging, all measuring < 7 mm. Ultimately, these results point to the effectiveness of contrast-enhanced MRI in the initial diagnosis of pituitary disorders in light of the evidence supporting non-contrast follow-up in stable conditions.

In this study, there were no significant differences in the size of the tumor. The temporal changes in the microadenoma’s size in both non-contrast and contrast images between primary and follow-up were comparable. This was compatible with the Hordejuk study, in which 62.7% of the microadenomas had no increase in size. Even when there was a growth, it was very slow. The authors suggest that infrequent pituitary MRI follow-up of patients with incidental pituitary microadenomas may be safer than regular contrast-enhanced follow-up [11]. The appropriate utilization of MRI monitoring in pituitary adenomas is critical to minimize healthcare expenses and decrease patient exposure to gadolinium. It can be suggested that follow-up guidelines be less strict for non-functioning microadenomas [12].

Our research included a larger group than previous studies conducted by other researchers [22,23]. Our study, however, has involved a follow-up MRI for the patients with pituitary adenoma, hence further improving the sample size, which, again, increases the generalizability of our findings. This adds to the available literature on this topic. 

Today, modern technology, such as AI-driven synthetic contrast (Tsui et al., 2024 [18]), provides new gadolinium reduction techniques; however, still, our non-contrast protocol provides distinct advantages for microadenoma follow-up in terms of cost-effectiveness and accessibility. A non-contrast MRI imaging requires USD 150–300 per scan without additional cost of contrast agents or AI software. AI synthetic contrast cost is almost USD 500–800 and it requires contrast-enhanced training scans plus computational substructures. Regarding accessibility, non-contrast MRI is universally available, even in resource-limited settings (1.5T scanners suffice); however, AI solutions require high-field MRI (3T) for training data and vendor-specific AI platforms (limited approvals).

Despite providing valuable insights, this study has some potential limitations. The retrospective nature of the analysis may introduce selection bias, and the variability in the MRI protocols across different institutions may affect the generalizability of the findings. Furthermore, reliance on radiologist interpretation may introduce variability in detection rates. While radiological stability is evident, the systematic hormonal follow-ups for 34/79 patients (43%) remain missing. Radiological stability does not necessarily afford hormonal stability, particularly in regard to functional adenomas. Our data gathered on the 1.5T scanner would not automatically generalize to 3T scanners, which may have better sensitivity for T2W. Patients undergoing DCE-MRI are probably those with worrisome presentations; therefore, it might be potential sampling bias.

There were some limitations regarding picture archiving and communication system (PACS) data, which lacks thorough clinical and hormonal examinations. Despite the absence of a priori power calculation in our study owing to retrospective design, post hoc analysis established sufficient power (92%) for detection of any clinically relevant difference in sensitivity. Future prospective validation studies will investigate cohorts of a larger size. Future studies must strive to include an even more comprehensive dataset, inclusive of patient-reported outcomes and other aspects of long-term follow-up data. Longitudinal studies tracking the natural history of tumor microadenomas could provide insights into the growth patterns and predictors of stability, thus informing clinical guidelines on monitoring and treatment initiation, as pointed out in recent reviews [12].

Moreover, further research into the relationship between molecular and genetic factors with pituitary adenomas would provide fresh insights into their pathophysiology. According to some research, there were specific mutations related to tumor behavior that created a possible consideration of targeted therapies and personalized management strategies [42,43].

## 5. Conclusions

The integration of non-contrast MRI into the initial diagnostic work-up for detecting microadenomas assumes great importance in light of our findings that microadenomas in our cohort have never increased in size. This has stressed the need for the detection of these lesions with non-contrast imaging, and shows that if they are reported properly by radiologists, there might not be a need for further assessments through dynamic contrast-enhanced imaging in follow-ups. Microadenomas detected in non-contrast sequences must be reported clearly and fully, supporting an argument for the possibility of diagnosing microadenoma, especially non-functional cases; therefore, non-contrast follow-up can be performed, which minimizes patients’ exposure to potential adverse reactions from contrast agents. This is a safety consideration for patients, and also reveals an intention to optimize the resource use within the healthcare system.

## Figures and Tables

**Table 1:** MRI pulse sequence parameters for T1, T2, and dynamic contrast-enhanced MRI (DCE-MRI). TR = time of repetition; TE = time of echo; FOV = field of view.

Sequence	TR/TE (ms)	Slice Thickness (mm)	FOV (mm)	Matrix Size	Resolution (mm^2^)
T1W	500/12	5	240	256 × 256	0.9 × 0.9
T2W	4000/100	5	240	256 × 256	0.9 × 0.9
DCE-MRI	500/12	5	240	256 × 256	0.9 × 0.9

**Table 2:** Frequency and percentage and 95% confidence interval of microadenoma detection in post-contrast, T2, and T1 sequences compared with dynamic images.

Imaging	Subcategory	Count #	Percent %	95% Confidence Interval	*p*-Value (vs. DCE-MRI)
Post-Contrast	Negative	9	11.3	[4.6%, 18.2%]	Reference
	Positive	70	88.6	[79.0%, 98.2%]	----
T2	Negative	23	29.1	[20.5%, 37.6%]	0.005
	Positive	56	70.9	[60.6%, 81.2%]	----
T1	Negative	35	44.3	[36.8%, 51.8%]	< 0.001
	Positive	44	55.7	[44.8%, 66.6%]	----

## Data Availability

The data presented in this study are available on request from the corresponding author due to confidentiality concerns.
